# Dispersion Process and Effect of Oleic Acid on Properties of Cellulose Sulfate- Oleic Acid Composite Film

**DOI:** 10.3390/ma8052346

**Published:** 2015-04-30

**Authors:** Guo Chen, Bin Zhang, Jun Zhao

**Affiliations:** Department of Biotechnology and Bioengineering, Huaqiao University, Xiamen 361021, China; E-Mails: zhangbinzbgg@126.com (B.Z.); zhaojun@hqu.edu.cn (J.Z.)

**Keywords:** packaging film, cellulose sulfate, oleic acid, composite films, hydrophobic properties

## Abstract

The cellulose sulfate (CS) is a newly developed cellulose derivative. The work aimed to investigate the effect of oleic acid (OA) content on properties of CS-OA film. The process of oleic acid dispersion into film was described to evaluate its effect on the properties of the film. Among the formulations evaluated, the OA addition decreased the solubility and water vapor permeability of the CS-OA film. The surface contact angle changed from 64.2° to 94.0° by increasing CS/OA ratio from 1:0 to 1:0.25 (w/w). The TS increased with OA content below 15% and decreased with OA over 15%, but the ε decreased with higher OA content. The micro-cracking matrices and micro pores in the film indicated the condense structure of the film destroyed by the incorporation of oleic acid. No chemical interaction between the OA and CS was observed in the XRD and FTIR spectrum. Film formulation containing 2% (w/w) CS, 0.3% (w/w) glycerol and 0.3% (w/w) OA, showed good properties of mechanic, barrier to moisture and homogeneity.

## 1. Introduction

Film and coating based on biomaterials as an alternative packaging draws lots of attention because of the consumers’ demand for high quality foods and increased awareness of environmental issues [1]. The commonly used biomaterials are comprised of proteins, polysaccharides, lipids, and other degradable biomaterials [2].

Cellulose is d-glucopyranose unit of conformation chair bonded through β (1→4) glycosidic linkages. Cellulose ethers are a class of semi-synthetic polymers obtained by derivatization of the hydroxyl groups at positions 2, 3, and/or 6 of the anhydroglucose residues of cellulose. Hydroxypropylmethyl cellulose (HPMC), carboxymethyl cellulose (CMC) and methyl cellulose (MC) as derivatives with improved solubility have long been used in fiber, film and gel-based materials [3]. Films made from cellulose derivatives showed good tensile resistance and effective barrier against O_2_/CO_2_ [4,5]. Nonetheless, edible films prepared from cellulose derivatives do not act as an efficient water vapor barrier due to the hydrophilic nature of cellulose [6,7,8]. Cellulose nanocrystals (CNC) synthesized from microcrystalline cellulose by a sulfuric acid hydrolysis was added to PLA or PLA–PHB film to improve the thermal stability of the film and reduce water permeability of the film [9,10]. One method to improve the water vapor barrier of cellulose films is incorporation of hydrophobic substances (fatty acids, beeswax, lipids) into the hydrocolloid matrix either by emulsification of hydrophobic substances and hydrocolloid aqueous solution before drying to obtain film, or the formation of bilayer films with a hydrophobic layer over the hydrocolloid based film. Many authors have studied the influence of hydrophobic substances addition on the properties of edible films [11,12,13,14,15]. Some researchers applied edible film based on composites of cellulose derivatives and hydrophobic substances to the fruit coating [16].

Cellulose sulfate (CS), prepared by partial or complete substitution of the 6-hydroxyl groups (–OH) with sulfate group (–SO_3_H), is a newly developed cellulose derivatives for several medical and biotechnological applications because of its biocompatibility and easy biodegradability [17,18,19]. According to our previous study [20,21], the film based on CS had poor water vapor barrier due to its excellent solubility. It is important to increase the water vapor barrier of the CS based packaging film for extending its application. Hydrocolloids films incorporation of lipid can result in better functionality than films of single component. Glycerol was also used in film as one of the most popular plasticizers used in film-making techniques, due to stability and compatibility with hydrophilic bio-polymer [22]. The objective of this work was to evaluate the influence of oleic acid incorporation into CS film on the mechanical, optical, structural, and water vapor barrier properties of CS films as compared with the pure CS films, using glycerol as plasticizer.

## 2. Results and Discussion

### 2.1. Rheological Behavior of the Film-Forming Emulsions

**Figure 1:**
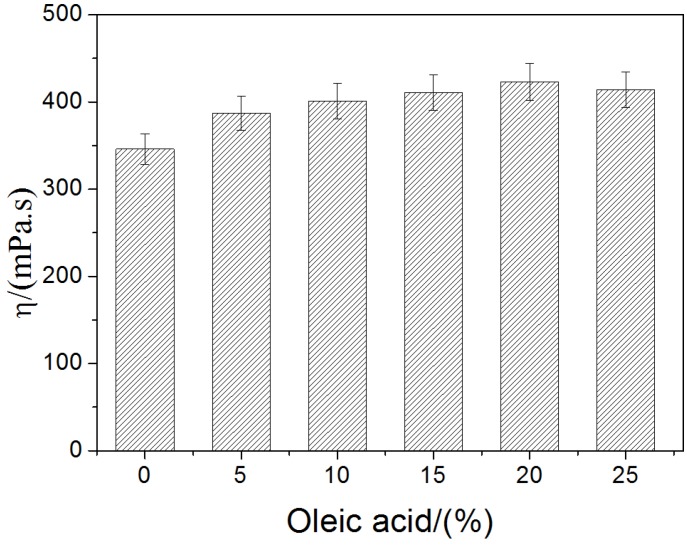
The effect of oleic acid on the viscosity of film forming emulsions.

**Graph 1:**
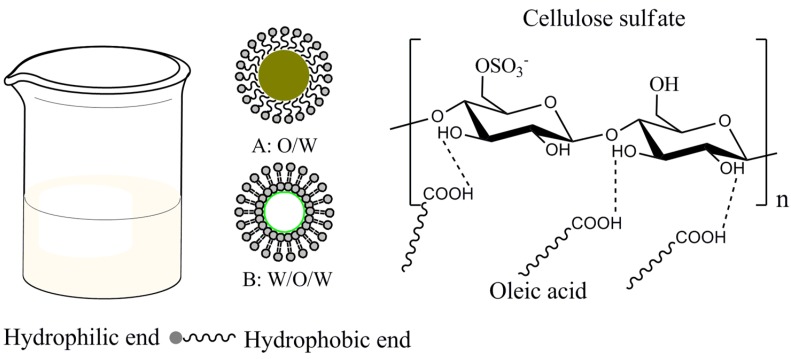
The schematic for emulsion of oleic acid, cellulose sulfate, glycerol and water.

The viscosity of film-forming solution is important to avoid non-uniform in thin liquid film after coating. As shown in Figure 1, the viscosity of the CS/OA blends increased from 346 mPa·s^−1^ to 423 mPa·s^−1^ when the OA content varied from 0% to 25%. The emulsion viscosity is influenced by various factors, mainly by the continuous phase viscosity, interfacial film viscosity and droplet size [23]. In this work, the interfacial film viscosity was same to the continuous phase viscosity, because all of the emulsions were prepared with the same CS content under the same homogenization conditions. The variation of emulsion viscosity was mainly attributed to oil droplet size caused by variation of OA content in emulsion and the homogenization velocity. The O/W and W/O/W were possible oil-in-water structure, but O/W was more stable. In O/W structure, hydrogen bond formed between carboxyl (–COOH) of oleic acid and hydroxyl (–OH) of cellulose sulfate as shown in Graph 1. Generally, the size distributions of oil droplet in O/W emulsions were trimodal with peak maximum, respectively, at 0.2 μm (I), 10 μm (II) and 100 μm (III), in which the predominance was population II according to previous research [23,24]. The number of droplet population increased with OA addition, which lead to the viscosity increasing due to the enhanced contact probability between O/W interfacial area and coaxial cylinder of viscometer. When OA content reached a critical value, the number of droplet do not increase any more at the same homogenization conditions, whereas the size of droplet increased due to the coalescence of droplets. Hence, the OA content over critical value would decrease the homogeneity of the O/W emulsion; correspondingly, the structure of the film became uneven.

### 2.2. Microstructure of CS-OA Films

**Figure 2:**
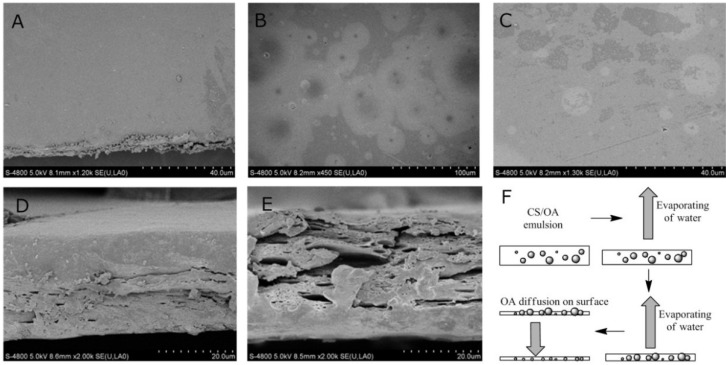
Surface appearance and cross-section appearance of films; surface of CS film (**A**); CS + 25% OA film (**B**); CS + 25% OA film (**C**) and cross section of CS film (**D**); CS + 25% OA film (**E**); and process of CS-OA film formation during water evaporating (**F**).

The morphology of the CS film and composite film (CS + 25% OA) are compared in Figure 2 to investigate their microstructure. The surface of CS film and composite film were smooth. The circle splotch of different size on the film surface in Figure 2B and C was observed. It should be the function of the OA diffusion on the surface. The splotch formation was accompanied with the CS-OA film formation during water evaporating, as shown in Figure 2F. During water evaporating, the oil droplet in film emulsion will move down with the liquid level. The intermolecular distance of CS becomes closer with the decreasing of water content in emulsion; the hydrogen bonds between CS molecules were strong enough to prevent the oil droplet moving in film emulsion. Pressure at O/W interface increased dramatically with the water continuous evaporation, especially near the interface of the film, correspondingly spherical droplet became ellipsoidal and oil accelerated diffusion into the film matrix over time, like a droplet of oil on paper. Though the number of 100-μm (III) droplets was small, it definitely diffuses at film surface, because its size was much bigger than thickness of the film. Therefore, more large-size splotches were observed in film surface, as shown in Figure 2. Fabra *et al.* [25] reported that the lipid particle size in the film emulsion containing ι-carrageenan, glycerol and lipids mixtures of oleic acid (OA)/beeswax (BW) varied from 4.3 to 20.3 μm with the ratio variation between OA and BW lipid. Nevertheless, Ghasemlou *et al.* [26] reported that the D_3,2_ (volume-surface mean diameter) of lipid particles in composite film emulsion based on kefiran and oleic acid (OA) varied from 1.13 μm to 2.06 μm. The results were quite different from each other. In our research, the lipid particle was not observed in the surface microstructure and the size of splotch on surface varied from ~10 to ~50 μm, according to Figure 2. Limpisophon *et al.* [27] also indicated that the films with oleic acid did not have crystalline particles like films with stearic acid, since oleic acid is in a liquid state at room temperature (m.p. = 13–14 °C) in edible films based on blue shark (*Prionace glauca*) skin gelatin. Cross-section images of CS films and CS-OA films with 25% OA are compared in Figure 2, in which the cross-sectional microstructure of CS-OA composite film was rougher than that of CS film. The CS-OA blend film also showed micro-cracking matrices and micro pores of different shapes and sizes, which meant the condense structure of film destroyed by the incorporation of oleic acid. The main pore size was 0.2 μm (I) according to Figure 2E, which was different from the droplet size in emulsion reported [23,24].

### 2.3. Surface Water Contact Angle of CS-OA Films

**Figure 3:**
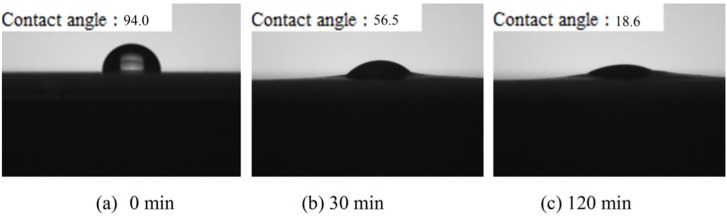
Surface water contact angle of oleic acid-cellulose sulfate films (CS + 25% OA film).

Contact angle is the angle (θ) between film surface and tangent at the droplet-film intersection. It can be used to indicate the hydrophobicity of the surface or the wettability of polymers [28]. The contact image between water droplet and CS-OA film at 0, 30, and 120 min is shown in Figure 3. The initial water contact angle for CS-OA film was 94°, whereas the water contact angle for pure CS film is 64.2°. Therefore the inclusion of OA in CS film increased the hydrophobicity. The droplet gradually permeated into film through the CS matrix. After 30 min the droplet left became small. After 120 min, some water unabsorbed was still observed on the surface of CS-OA films, compared with soluble hole formed on the film surface after 60 s for pure CS film [21]. This indicated that CS-OA film was less rapidly wetted, which explained the decreasing water solubility of CS-OA films as shown in Table 1.

### 2.4. The Effect of Oleic Acid Content on Properties of CS-OA Films

Some properties of CS-OA films with different OA content, such as the thickness, flexibility, integrity, water solubility, oil permeability, transparency, mechanical properties and water vapor permeability are shown in Table 1. The integrity and folding property of all films are good, which was consistent with their easy peeling from the Teflon surface. The thickness, solubility and oil permeability of the CS-OA films did not change significantly under different OA content. The solubility and the transparency of composite films decreased gradually with addition of OA in film-forming emulsion, correspondingly the thickness of composite films and oil permeability increased with the OA content. The increasing of oil permeability can be attributed to the tunnel provided by the OA in the film.

**Table 1:** The effects of OA content on properties of oleic acid-cellulose sulfate films.

Sample	δ (μm)	Inte	Fold	S (h)	OP (%)	T (%)	TS (MPa)	ε (%)	WVP × 10^−11^ (gm^−1^s^−1^Pa^−1^)
2g CS + 0.3g Gly	25.00 ± 0.16 ^a^	good	good	0.02 ± 0.01 ^a^	0.2 ± 0.1% ^a^	89.3 ± 3.2 ^a^	14.5 ± 1.5 ^a^	27.9 ± 0.8 ^a^	3.92 ± 0.23 ^a^
2g CS + 0.3g Gly + 0.1g OA	25.02 ± 0.10 ^a^	good	good	1.50 ± 0.10 ^b^	0.2 ± 0.2% ^a^	85.0 ± 5.6 ^a^	28.2 ± 2.1 ^b^	18.6 ± 0.6 ^b^	3.49 ± 0.21 ^a^
2g CS + 0.3g Gly + 0.2g OA	25.85 ± 0.18 ^b^	good	good	2.33 ± 0.08 ^c^	1.9 ± 0.2% ^b^	76.9 ± 2.8 ^b^	37.1 ± 1.7 ^c^	13.9 ± 0.3 ^a^	2.41 ± 0.16 ^b^
2g CS + 0.3g Gly + 0.3g OA	29.04 ± 0.12 ^c^	good	good	4.45 ± 0.12 ^d^	9.0 ± 0.3% ^c^	73.5 ± 4.8 ^b^	43.5 ± 1.3 ^d^	9.2 ± 0.5 ^d^	1.91 ± 0.12 ^bc^
2g CS + 0.3g Gly + 0.4g OA	36.60 ± 0.26 ^d^	good	good	5.13 ± 0.50 ^de^	14.6 ± 0.5% ^d^	67.4 ± 2.3 ^c^	36.8 ± 1.5 ^e^	7.2 ± 0.3 ^e^	1.57 ± 0.21 ^c^
2g CS + 0.3g Gly + 0.5g OA	55.20 ± 0.32 ^e^	good	good	5.55 ± 0.36 ^e^	25.1 ± 0.8% ^e^	68.2 ± 3.1 ^c^	34.3 ± 2.2 ^e^	6.4 ± 0.5 ^e^	1.52 ± 0.14 ^c^

δ: thickness; Inte: integrity; Fold: folding properties; S: solubility time; OP: oil permeability; T: transparency; TS: tension strength; ε: elongation at break; WVP: water vapor permeability; Mean ± standard deviation. Different letters represent significant differences (*p* < 0.05) according to the LSD test.

The ability of the coating to form a continuous layer over the product and the durability of the film are important, which can be reflected partly by mechanical properties listed in Table 1. The TS increased, but the ε decreased, when increasing OA content below 15%. The high TS and low ε indicated stronger and less extendible films. OA content over 15% decreased the values of all mechanical parameters, forming weaker and less extendible films. Elongation of CS-OA film significantly (*p* < 0.05) decreased when oleic acid was incorporated into the CS matrix, which has already been reported in other hydrocolloids films containing lipids [29,30,31]. It was explained by the fact that lipids were unable to form a cohesive and continuous matrix in the film. Oleic acid has also been reported to increase elongation of soy protein, corn zein, egg white and HPMC films [13,32,33]. They explained this as a plasticizing effect of unsaturated oleic acid. The effect of lipids on mechanical of hydrocolloids film may be dependent on the basic matrix properties of biomaterials, the interaction of the polymer molecular, the component of the film and the size distribution of the lipid droplet.

The WVP values of the CS-OA emulsified films are presented in Table 1. As expected, the water vapor permeability decreased when the OA content increased. Incorporation of fatty acids caused a significant difference (*p* < 0.05) between the WVP of the CS films containing different OA content. The WVP of hydrocolloids–fatty acid films decreased as the content of fatty acids increased. The WVP decreased from 3.92 × 10^−11^ to 1.52 × 10^−11^ gm^−1^s^−1^Pa^−1^ as OA content reached 25% of the CS in the CS-OA film. The OA dispersed in the CS film decreased the practical interfacial area exposed to water vapor. In general, the relative polarity of the support polymer and the type of lipid has the strongest influence on the water vapor barrier of emulsified films. Similar results were obtained by other researches [34,35,36]. However, as the amounts of fatty acids increased from 20% to 25%, no significant differences (*p* > 0.05) in the WVP values among the emulsified films were observed. Fabra *et al.* [37] showed that when the beeswax content added came to over 30% of the total lipid phase (70:30 OA:BW relationship) no further reduction in the WVP of sodium caseinate films was observed. The poor dispersion of lipid in the film system with the increasing lipid content may be account for the result. Size, distribution and physical state of the lipid, and polymorphism also seem to play a role in WVP, especially when the lipid content is over a critical value.

### 2.5. The Interactions among Components of Edible Film

**Figure 4:**
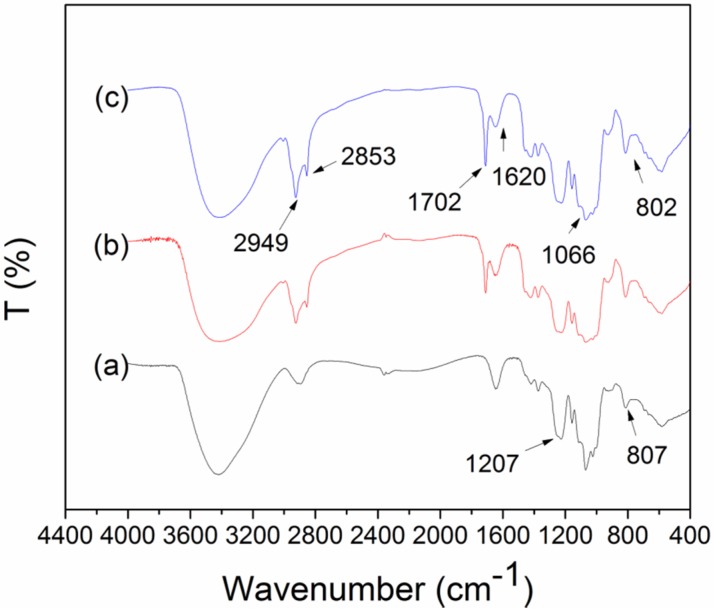
FTIR spectra of CS-OA films (a) 0%; (b) 10%; (c) 25% OA.

When two or more substances are mixed, physical blends *versus* chemical interactions can be reflected by the changes in characteristic spectra peaks [38]. FTIR spectra of the CS film with different OA content are shown in Figure 4. The assignments proposed for the bands observed were annotated. The strong broad band observed in the 3500–3000 cm^−1^ range was attributable to hydrogen bond between different O–H groups from OH of glucosidic ring, OH carboxylic function of oleic acid acids and OH of glycerol. A strong broad band at 1207 cm^−1^ was attributed to the –O-SO_3_^−^ stretching mode. A strong and composite band with a maximum intensity at 1066 cm^−1^ was assigned to the stretching mode of the C–O bond. A medium weak band at 2949 cm^−1^ was assignable to C–H stretching mode, which was more intense for CS-OA film than that for pure CS film. Several medium bands in the region 1400–1200 cm^−1^ was assigned to the C-H bending and wagging modes, and O–H bending mode. A weak band at 807 cm^−1^ was assigned to the stretching mode of the glucosidic ring from CS. The new frequency at 2853 cm^−1^ for CS-OA film was attributed to the C–H stretching mode of the aliphatic chain of the fatty acids. The intense IR band at 1702 cm^−1^ and 1620 cm^−1^ corresponded to C=O and COO^−^ stretching mode of oleic acid, respectively. These characteristic peaks of fatty acids only appeared in the CS-OA composite films. No additional chemical grouping was found on the FTIR spectra, which indicated no chemical bond formed between the lipid and CS.

**Figure 5:**
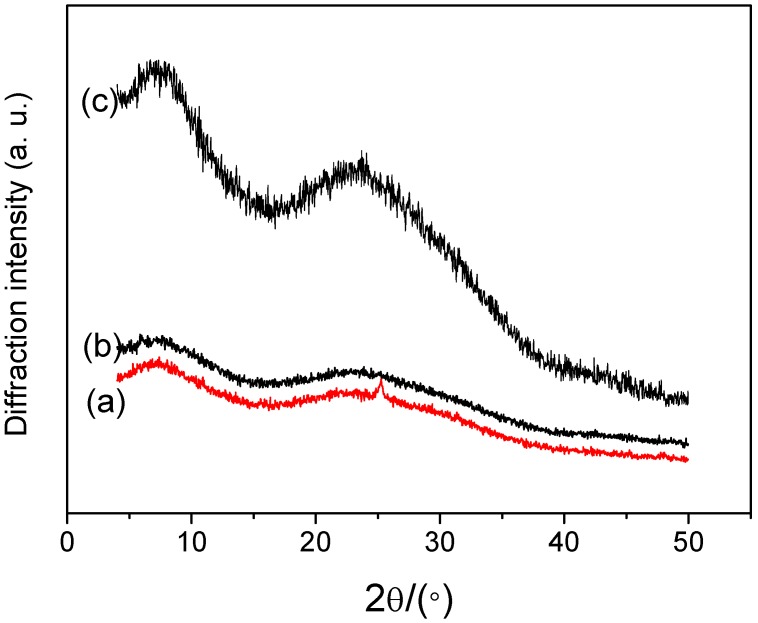
XRD patterns of CS-OA films (a) 0%; (b) 10%; (c) 25% OA.

X-ray diffractogram (XRD) was used to investigate crystal structure, and assess the compatibility of CS and oleic acid. XRD of CS film with different contents of oleic acid blended were measured as shown in Figure 5. The XRD of CS film and CS-OA film showed two main crystalline reflections at 7.9° and 23.1°, presenting the characteristic of major amorphous structures for all films, which was similar with the fine structure of CMC [39]. No new diffraction peaks were observed in composite films, suggesting no intermolecular interactions between CS and OA.

## 3. Experimental Section

### 3.1. Materials

Cellulose sulfate (CS) was synthesized using heterogeneous method [17], with dynamic viscosity (η_2%_, 2 wt % solution) of 346 mPa·s^−1^ and average substitution degree of 0.4. Glycerol and oleic acid (OA, C18:1) were purchased from Sinopharm Chemical Reagent Co., Ltd. (China). Distilled water was used to form samples preparation.

### 3.2. Preparation of Cellulose Sulfate Films

Four different formulations based on cellulose sulfate (CS), glycerol (Gly) and oleic acid (OA) were prepared. CS (2 g) and Gly (0.3 g) were dispersed in 100 mL water in order to obtain polysaccharide dispersions. The OA fraction was incorporated in a CS–OA ratio of 1:0.05, 1:0.10, 1:0.15, 1:0.2, 1:0.25 and the mixture was homogenized at 13,000 rpm for 1 min, under vacuum, using a rotor-stator homogenizer (Ultraturrax T25, Janke and Kunkel, Germany). Then the film forming dispersions were gently spread over a leveled Teflon plate (150 mm diameter, Wei Xin Instrument Co., Ltd., Yixing, China) with 0.18 g cm^−2^ and dried for approximately 4 h at 60 °C and 45% RH. Afterwards, the films were peeled from the casting surface and stored in desiccators at 75% RH for further testing. All treatments were made in triplicate.

### 3.3. Rheological Behavior of the Film Forming Emulsions

The dynamic viscosity of the film-forming solution was measured at 30 ± 0.5 °C with a NDJ-5S rheometer (model NDJ-5S, Fangrui Instrument Co. Ltd., Shanghai, China). The range of shear rate, 100–300 s^−1^, was used because it covered all the concentrations of the samples using the same coaxial cylinder device. Each sample was analyzed in triplicate.

### 3.4. Fourier Transform Infrared Spectroscopy (FTIR)

The FTIR transmission spectra of the film prepared was recorded on an instrument (Shimadzu FTIR 8400S, Kyoto, Japan) in the wavenumber range of 4000–400 cm^−1^, using Attenuated Total Reflectance mode (ATR). Spectra were recorded at a resolution of 4 cm^−1^ and 400 scans were carried out to obtain a high signal-to-noise ratio spectrum.

### 3.5. X-ray Diffraction

X-ray diffraction (XRD) patterns of all samples were analyzed by a X'Pert PRO XRD system (PANalytical, Almelo, The Netherlands) at 25 °C and 75% RH, between 2θ = 10° and 2θ = 80° using Kα Cu radiation (λ = 1.542 Å), 40 kV and 40 mA with a step size of 4°. Samples were cut into 2 cm squares for analysis.

### 3.6. Film Thickness

The thickness (δ) of the sample was measured (exactness of ±0.001 mm) by a digital external micrometer (Mitutoyo Co., Tokyo, Japan) at ten different points of the film. Samples were conditioned at 25 °C and 75% RH (a saturated NaCl solution) for 24 h before they were measured.

### 3.7. Scanning Electron Microscopy

The samples were maintained in a desiccator with P_2_O_5_ for two weeks to ensure water in films completely removed. The cross-sections of the films were observed by cryofracture of films frozen in liquid N_2_. The microstructure of the film was analyzed using a scanning electron microscope (Hitachi S4800, Tokyo, Japan). Samples were fixed on a copper stubs, gold coated, and observed using an accelerating voltage of 5 kV.

### 3.8. Mechanical Properties

Tensile strength (TS) and elongation at break (ε) of the film were measured using a Instron Universal Testing Machine (Instron Corp., model 5569, MA, USA) according to the standard method [40]. Test samples, 25 mm × 100 mm, were cut from each film and fixed with an initial grip separation of 30 mm. Five replicates of each film were then pulled apart at crosshead speed of 20 mm/s and preload of 2 N. The average thickness of film was 25 ± 2 μm. TS (MPa) was calculated by the Equation (1):

TS = F_max_/A (1)

where F_max_ is the maximum force (N) loaded on the specimen before pulling apart; A is the cross-sectional area (m^2^) of the specimen. εis defined as the Equation (2):

ε = ∆L/L_0_ × 100% (2)

where ∆L is the film elongation at the moment of rupture (mm) and L_0_ is the initial length between the grips.

### 3.9. Water Vapor Permeability (WVP)

WVP data were measured according to the standard ASTM method (1995) [41]. Test samples, 90 × 90 mm, were cut from each film and sealed on cups which was previously filled with fused anhydrous CaCl_2_ (RH = 0%). And then the cups were placed into a humidity chamber at 25 °C and 75% RH (saturated NaCl solution) for 3 days. The sealed cups were weighed periodically (±0.0001 g) to calculate water vapor transported into the cup. The data, weight *vs.* time, was linearly regressed to calculate the slope. The water vapor transmission rate (WVTR) through the film was calculated from the slope (Δw/Δt) of the fitted line divided by the test area (A) as Equation (3), (g s^−1^m^−2^).

WVTR = Δw/(Δt·A)
(3)

where w is the weight of water transported into the cup (g), t is the time for weight change (s), A is the area exposed to water vapor transfer (m^2^). The WVP (gm^−1^s^−1^Pa^−1^) is calculated as Equation (4).

WVP = (WVTR × δ)/Δp
(4)

where δ is the film thickness (m) and Δp is the water vapor partial pressure difference across the two sides of the film (Δp = p(RH_2_ − RH_1_) = 2081.325 Pa, where p is the saturation vapor pressure of water at 25 °C, RH_2_ = 75%, RH_1_ = 0%) (Pa).

### 3.10. Contact Angle

Contact angle of the film was measured using a Video-Based Contact Angle Meter model OCA 20 (DataPhysics Instruments GmbH, Filderstadt, Germany). A droplet of 3 μL ultrapure water was dispensed on each film surface using a micro syringe. The contact angle was recorded by analyzing the shape of a sessile drop after it had been placed over the surface of each film at different time. Image analysis was carried out by SCA20 software. Each sample was tested with three drops and three measurements were conducted for each water drop.

### 3.11. Flexibility

The flexibility of the film was determined by a bending method [21]. Each sample was cut into the size of 20 × 40 mm and folded completely at the middle. The film was positively and negatively folded in turn until the film appeared rupture. The flexibility was rated as poor (folding number < 20), middle (20 ≤ folding number < 50), good (50 ≤ folding number < 100) and excellent (folding number ≥ 100).

### 3.12. Integrity

The CS film was deemed as good integrity of there was no breakage during peeling from the petri dish and *vice versa*.

### 3.13. Oil Permeability

Oil permeability of the film was determined following Hu’s method [42]. The mouth of a glass test-tube filled with 3 g soybean oil (interior diameter: 25 mm, and outer diameter: 27 mm) was covered with CS film (50 × 50 mm) and sealed tightly. The tube was upside down on a piece of filter paper. The oil penetration was judged according to the infiltration of soybean oil on filter paper as time going. Each sample was observed in triple.

### 3.14. Water Solubility

The solubility of film in water was characterized by the resolve time of the film. The test sample was placed into deionized water at 25 °C until it was completely dissolved. Three samples of each film were tested.

### 3.15. Transparency

Film specimen was cut into a rectangle piece (20 × 10 mm) and attached on the wall of a test cell directly. The light transmission (T %) of sample was measured at 560 nm using a UV-722 spectroscope (Rayleigh Corp., model 722, Beijing, China) and air was used as the reference. Three samples of each film were tested.

### 3.16. Statistical Analysis

Data for each test were are presented as mean ± SD after statistical analysis. The significance in the difference between factors and levels was evaluated by the analysis of variance (ANOVA). Comparison of the means was done employing a Tukey test to identify which groups were significantly different from others (*p* < 0.05).

## 4. Conclusions

The above findings indicated that the hydrophobicity of CS films can be regulated by incorporation of OA. The OA incorporation into CS film decreased the cohesive matrix of the film, which was severely affected by the size distribution of the OA droplet in film-forming solution. The solubility and transparence of the CS-OA decreased, while the thickness and oil permeability dramatically increased with the OA content increasing. Films with the addition of OA presented better water vapor barrier properties as compared to pure CS films. The WVP decreased from 3.92 × 10^−11^ to 1.45 × 10^−11^ gm^−1^s^−1^Pa^−1^ when OA content varied from 0 to 30%. The TS and E% of the CS-OA film decreased, which indicated the film was more fragile than pure CS film. Films with a 1:0.15:0.15 CS:glycerol:OA ratio showed the most adequate functional properties when considering both tensile and water transport properties (TS: 43.5 ± 1.3 MPa, E%: 9.2 ± 0.5, WVP: 1.91 ± 0.12 × 10^−11^ g m^−1^s^−1^Pa^−1^).
